# Pomarius

**Published:** 1875-10

**Authors:** 


### THE HEALTH FOOD COMPANY.

POMARIUS.—The Health Food Company have a delicious fruit product which ought to be in every household, and on every table in the land, at least once each day. If it were moderately eaten daily, we should hear far less of consumption, torpid livers, and dyspepsia. We must have some fruity sub-acid if we expect to have the best and most healthful condition of the alimentary canal. This new product hits the case precisely; it is the filtered juice of sound, mellow, tart apples, concentrated 90 per cent.—that is, from 100 volumes to 10 volumes. It never ferments, and would, no doubt, keep for ages. There is no sugar in it, except such as the juice contained. Everybody calls it delicious, and children literally cry for it. Unlike many things for which they cry, their desire in this regard may be safely gratified to any reasonable extent.

We take pleasure in calling attention to the advertisement in another column of Van Buskirk's Fragrant Sozodont. There is no more delightful and refreshing article known for preserving and polishing the teeth than this. It is also an excellent article for protecting the deciduous teeth of young children, till they are replaced by the permanent ones. Try it, and you will never be without it.—*Methodist, May* 29, 1875.

